# INPP4B inhibits glioma cell proliferation and immune escape *via* inhibition of the PI3K/AKT signaling pathway

**DOI:** 10.3389/fonc.2022.983537

**Published:** 2022-09-06

**Authors:** Xiaoming Sun, Yani Chen, Xiaoyang Tao, Wenzi Zhang, Xinyu Wang, Xianhui Wang, Zhihua Ruan, Zhuo Chen

**Affiliations:** ^1^ School of Basic Medical Sciences, Hubei University of Medicine, Shiyan, China; ^2^ Biomedical Research Institute, Hubei University of Medicine, Shiyan, China; ^3^ Hubei Key Laboratory of Embryonic Stem Cell Research, Hubei University of Medicine, Shiyan, China; ^4^ Department of Anesthesiology, Taihe Hospital, Hubei University of Medicine, Shiyan, China

**Keywords:** INPP4B, glioma, immune escape, PI3K/AKT signaling, PD-L1

## Abstract

INPP4B (Inositol polyphosphate 4-phosphatase type II) has been regarded as a suppressor of several human tumors, but its biological function, expression, and clinical significance in glioma tissues and cell lines are unclear. Notably, whether INPP4B participates in immune escape of glioma deserves urgent attention. Here, we confirmed that INPP4B expression is often downregulated in low- and high-grade human glioma tissues, in tissues from an orthotopic mouse model of brain glioma and in glioma cells. We found that INPP4B overexpression restrained the proliferation, migration, apoptosis resistance, PD-L1 expression, and T cell suppression by glioma cells, whereas INPP4B silencing had the opposite effects. Moreover, we showed that INPP4B inhibited glioma cell proliferation, migration, and PD-L1 expression by downregulating PI3K/AKT signaling. Collectively, these data support that INPP4B may inhibit glioma progression, and particularly, glioma’s immune escape. Thus, INPP4B may constitute a valuable target for glioma treatment.

## Introduction

Glioma is one of the most harmful malignant tumors in humans, accounting for about 50–60% of all intracranial primary tumors, among which glioblastoma (GBM) has the highest degree of malignancy. Typically, GBM is characterized by abnormal and rapid proliferation, extremely high invasiveness, and high heterogeneity, leading to a median survival of about one year only. Therefore, new treatments are urgently needed (1–4). The number of studies on new molecular therapies for glioma is rapidly increasing. Targeted therapies, still at early stages of clinical research, carry great promises. However, most patients who respond to primary targeted therapies eventually relapse because of the emergence of acquired drug resistance (5–7). Therefore, it is crucial to clarify the molecular mechanisms that promote the development and progression of glioma and develop effective therapeutic strategies.

Molecular studies have uncovered many genetic or epigenetic abnormalities and key signaling pathways involved in glioma growth (8–10). Inositol polyphosphate 4-phosphatase type II (INPP4B) is a phosphoinositide phosphatase that dephosphorylates phosphatidylinositol 3,4-bisphosphate [PI(3,4)P2] into phosphatidylinositol 3-phosphate [PI(3)P]. It is considered a tumor suppressor, as low expression of INPP4B can promote tumor growth through activation of the PI3K/AKT pathway (11–13). Downregulation of the PI3K/AKT pathway can restrain the expression of a variety of downstream molecules, such as Cyclin D1 and Bcl-2, thereby inhibiting the proliferation and enhancing the apoptosis of glioma cells (14). Recent studies found low INPP4B expression in gastric cancer, breast cancer and other tumor tissues (11–13), but there are few data on glioma. INPP4B and its control on PI3K/AKT pathway may play a critical role in the development of glioma.

PD-L1 is highly expressed in a variety of malignant tumors. By activating the PD-1/PD-L1 signal pathway, PD-L1 suppresses immune cells’ surveillance and cytotoxic activity, promoting the occurrence, development, and escape of malignant tumor cells, such as lung, liver, and breast cancers (15–17). Human glioblastoma is a typical tumor with immunosuppressive functions, including the production of transforming growth factor-beta (TGF-β) and IL-10. Some studies also showed that the expression of PD-L1 in glioma correlates with disease stage, according to the WHO grading, and may be considered as a tumor biomarker. PD-L1 contributes to protect glioma cells by inhibiting T-cell anti-tumor activity and strengthening Tregs functions, which prevent immune hyperactivations and limit T-cell responses (18–20). By increasing PD-L1 expression by the tumors, the loss of the tumor suppressor gene phosphatase and tensin homolog (PTEN) may play a role in tumor development and evasion (21, 22). Similarly, the tumor suppressor gene INPP4B, which shares common features with PTEN, may play a role in PD-L1 regulation and deserves further study (23, 24).

Interestingly, data derived from pre-clinical mouse models and clinical trials showed that inhibition of the PI3K-AKT signaling pathway could increase tumor immunosurveillance by improving immune anti-tumor properties and prevent immunosuppression. Studies found that silencing PTEN increased PD-L1 expression by glioma cells and implicated PI3K/AKT signaling in this pathway (18, 25). Other studies in a model of pancreatic cancer revealed that the blockade of PD-L1 had an antitumor and antimetastatic effects by regulating PI3K/AKT signaling (26). Altogether, these data support that inhibition of the PI3K/AKT pathway reduces the expression of PD-L1, and thus, prevent tumor evasion.

Here, we confirmed that INPP4B expression is markedly diminished in glioma compared with healthy brain tissues. We found that INPP4B overexpression restricted the proliferation, migration, apoptotic resistance, and PD-L1 expression by glioma cells, thereby inhibiting immune escape. Moreover, these effects were all due to the downregulation of PI3K/AKT signaling. These new findings bring new insights for potential therapeutic targets to restrict the progression of glioma.

## Materials and methods

### Cell culture

Human U87, U251, U373, and GL261 glioma and HA1800 normal astrocyte cell lines were stored in our laboratory. The T-cell lymphoma cell line Jurkat was obtained from ATCC (American Type Culture Collection). Mouse primary astrocytes were collected from the cerebral cortices of 3- to 4-day-old newborn Balb/c mice, according to published protocols, with slight modifications (27). The Jurkat cells were cultured in RPMI-1640 supplemented with 25 mM HEPES and 25 mM NaHCO_3_. All glioma cell lines and primary cells were cultured in DMEM/F12 medium (Gibco) supplemented with 10% fetal bovine serum (FBS; QmSuero/Tsingmu Biotechnology, Wuhan) in a humidified incubator at 5% CO_2_ and 37°C.

### Plasmid construction and transfection

A full-length cDNA encoding the human INPP4B protein was amplified from a vector containing the INPP4B cDNA, and then was cloned into the pcDNA3.1 expression vector. For transfection, U87 or U251 cells were seeded in 6-well plates and cultured in medium for 24 hours. When the culture reached 70–80% confluence, they were transiently transfected with the indicated vectors in Lipofectamine^®^ 2000 following the manufacturer’s instructions. An empty pcDNA3.1 vector and pcDNA3.1-scramble were used as the negative controls. In addition, INPP4B-specific siRNA (siRNA-INPP4B: 5’-AGUACAUACAGCGAUGAAAUUGGAA-3’) or negative control (siRNA-scramble: 5’-UUCUCCGAACGUGUCACGU-3’) were transfected into cells.

### RNA extraction, cDNA synthesis and quantitative real-time PCR

For INPP4B expression analysis, total RNA was isolated from the cells using TRIzol reagent (Ambion, USA) and was retrotranscribed into cDNA with a PrimeScript RT reagent (Takara, Shiga, Japan) as recommended by the manufacturer. For cDNA amplification, the primers specific for INPP4B were: 5’-GGAAAGTGTGAGCGGAAAAG-3’ (forward); 5’-CGAATTCGCATCCACTTATTG-3’(reverse). The primers to amplify the reference control cDNA corresponding to the GAPDH gene were: 5’-GGAGTCCACTGGCGTCTTCA-3’(forward) and 5’-GTCATGAGTCCTTCCACGATACC-3’(reverse). The PCR reaction was performed using a TB Green Fast qPCR Mix (Takara) following the manufacturer’s instructions using a CFX96 Touch Real-Time PCR Detection System (Bio-Rad, USA). Each test was performed in triplicate and the data were analyzed with the 2^-△△CT^ method.

### Western blot assay

Total protein was extracted from cell lines using RIPA Lysis Buffer (Biosharp, China) containing a protease inhibitor and quantified using a BCA Protein Assay Kit (Biosharp, China) according to the manufacturer’s instructions. Western blotting was performed using a standard protocol. The primary antibodies were: anti-INPP4B (BM4609, Boster, USA), anti-PD-L1 (66248-1-lg, Proteintech), anti-p85 (660225-1-lg, Proteintech), anti-Phospho-AKT (66444–1-lg, Proteintech), anti-Bcl-2 (12789–1-AP, Proteintech), and anti-Cyclin D1 (60186–1-lg, Proteintech). In addition, an anti-GAPDH (60004–1-lg, Proteintech) was used as loading control.

### Transwell migration assay

Transwell inserts (8 μm, 24-well format; Corning) were used to assess cell migration. Twenty-four-well plates were filled with 0.6 mL RPMI1640 supplemented with 10% fetal bovine serum in their lower chamber and were loaded with 5×10^4^ U87 or U251 cells transfected with pcDNA3.1-INPP4B or siRNA-INPP4B in 0.2 mL serum-free medium in their upper chambers. After incubation for 48 hours, the chambers were removed and washed twice with pre-cooled PBS. The remaining cells were fixed with paraformaldehyde for 10 min, stained with 0.1% crystal violet solution for 20 min, and photographed under a microscope.

### Apoptosis and flow cytometry

U87 and U251 cells were seeded in 12-well plates and transfected with either INPP4B-overexpressing plasmids or with siRNA-INPP4B for 48 h. Cells were then harvested, washed twice in PBS, and stained using an Annexin V-FITC reagent (meilunbio, China) at room temperature in the dark for 15 min. The percentage of apoptotic cells was measured by flow cytometry. The samples were analyzed using a BECKMAN COULTER CytoFLEX flow cytometer. Excitation laser wavelength was 488 nm. All data were acquired on a log scale. At least 10,000 events per sample were recorded for single-culture experiments and 20,000 events per sample were recorded for mixed-culture experiments. Geometric mean (GM) of each sample was calculated to quantify fluorescence intensity.

### Jurkat cell proliferation assay

The Jurkat cell proliferation assay was performed according to the protocol provided in the carboxyfluorescein succinimidyl ester (CFSE) kit. Briefly, 2 × 10^5^ CFSE-labeled Jurkat cells were added to the wells with 4×10^4^ U87 cells (Jurkat cells: Glioma cells = 5:1). The U87 cells had been transfected with pcDNA3.1-scramble or pcDNA3.1-INPP4B. The proliferation of the Jurkat cells was analyzed by flow cytometry. PD-L1 blockade was achieved by addition of a functional-grade anti-human PD-L1 antibody (10 µg/mL) to the glioma cell culture, which was incubated for 4 h before adding CFSE-labeled Jurkat cells.

### Specimen collection and immunohistochemistry

Twenty-five glioma specimens were collected by surgical resection and served to confirm the pathological diagnosis. In addition, 20 normal brain tissue samples from contused brain tissues removed during the operation of the patients with brain trauma were collected as control. All the samples were obtained with written informed consent and analyzed anonymously. The collection and use of clinical specimens for this study were approved by the Ethics Committee of Hubei University of Medicine. The glioma specimens were from 10 male and 15 female patients and included 12 low-grade and 13 high-grade gliomas. The pathological classification was performed according to the 2016 World Health Organization classification of tumors of the central nervous system. Paraformaldehyde-fixed paraffin tissues were stained by immunohistochemistry (IHC).

### Animal model

All experimental procedures and animal handling were carried out in accordance with the protocol approved by the animal care committee of Hubei University of Medicine.

Four- to five-week-old athymic BALB/c nude female mice, weighing 18–20 g, were purchased and used to construct an orthotopic GBM xenotransplantation model as previously described (27). Briefly, U87 cells (5 × 10^5^ cells/10 μL PBS) were injected into the right corpus striatum of each mouse slowly. Three weeks later, the animals were sacrificed, and the brains were harvested. Paraffin sections (5 μm) were prepared for IHC staining with anti-INPP4B, anti-p-AKT, anti-Cyclin D1, and anti-Bcl-2 antibodies.

### Statistics

Where applicable, all above quantitative data are expressed as means ± standard deviation (SD). Two-group comparisons were statistically evaluated by independent Student’s t-test, and three or more group comparisons were assessed by one-way analysis of variance (ANOVA) for statistical significance. The clinicopathological characteristics of the patients with glioma and INPP4B status were assessed by Fischer’s test and Pearson chi-square test, respectively.

## Results

### INPP4B expression is frequently diminished in glioma tissues and cell lines

**Figure 1:**
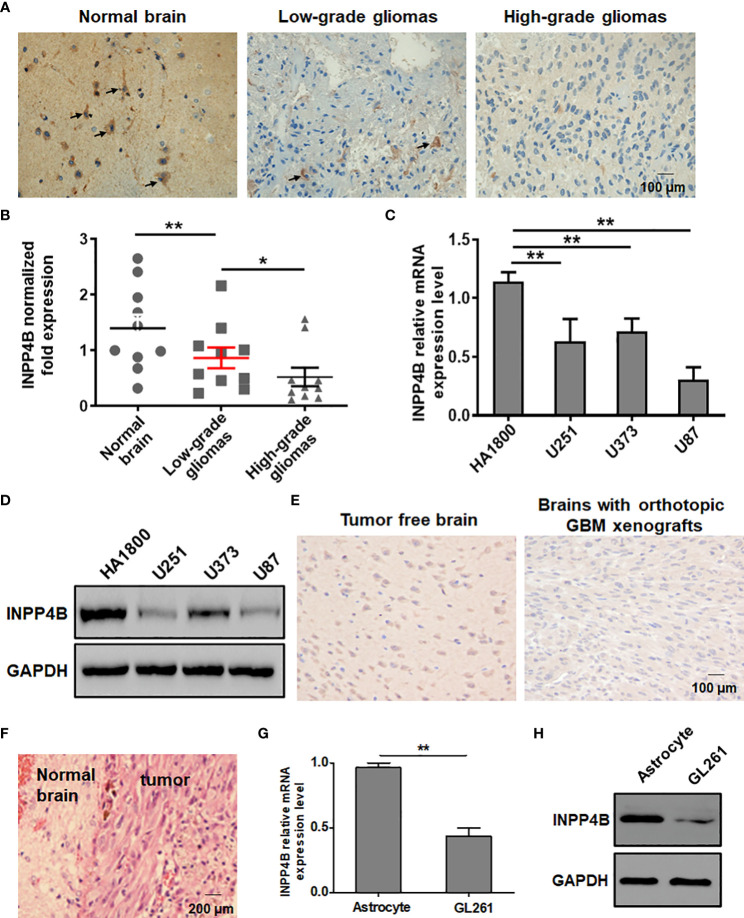
INPP4B expression is diminished in glioma tissues and cell lines. **(A)** IHC analysis of INPP4B expression in representative samples of normal brain tissues, and low- and high-grade glioma tissues. **(B)** Comparison of *INPP4B* mRNA levels in normal brain tissues (n = 10), low-grade glioma tissues (n = 10), and high-grade glioma tissues (n = 10). The relative abundance of *INPP4B* mRNA was calculated by reference to the amount of I*NPP4B* mRNA in normal brain tissues, arbitrarily assigned a value of 1. **(C)** Expression of *INPP4B* mRNA in a non-tumoral human astrocyte line (HA1800) and in three human glioma cell lines (U251, U373, and U87) assessed by qRT-PCR. **(D)** Western blot analysis of INPP4B protein expression in the same human cell lines as in **(C)**. **(E)** IHC analysis showing INPP4B expression in mouse orthotopic GBC xenografts brain tissues in comparison with normal brain tissues. **(F)** HE staining on a representative brain section from a mouse in which an orthotopic glioma xenograft was established successfully. **(G, H)** Expression of INPP4B in GL261 glioma cells and normal mouse astrocytes, assessed by qRT-PCR or western blot. Values represent the means ± SD (n = 3); *p < 0.05, **p < 0.01. Scale bars, 100 μm.

To probe a potential difference in INPP4B expression between normal brain tissues and glioma tissues, samples of normal brain tissue, low-grade and high-grade glioma tissues were analysed by IHC. INPP4B staining was predominantly located on the cell membrane and in the cytoplasm (**Figure 1A**). The level of INPP4B expression was evidently higher in normal brain tissues than in low- and high-grade glioma tissues (**Figure 1B**, **Table S1**). In addition, we verified that INPP4B was expressed at higher levels in low- than in high-grade glioma samples (**Figures 1A, B**). INPP4B expression was significantly and inversely correlated with histological grades (Table S2). These results were confirmed in human cell lines. INPP4B mRNA and protein levels were significantly lower in glioma cell lines (U251, U87, U373) than in the astrocyte cell line HA1800 (**Figures 1C, D**).

We sought to confirm these observations in a mouse model. To this aim, we constructed an orthotopic brain glioma model and confirmed its successful establishment by histology using HE staining of brain sections from the mice (**Figure 1F**). IHC analysis showed higher INPP4B expression in normal mouse brain tissues than in tumor-burdened brain tissues (**Figure 1E**). Further, INPP4B mRNA and protein levels were significantly lower in the murin glioma cell line GL261 than in primary astrocytes (**Figures 1G, H**).

Altogether, these data suggest that impaired INPP4B expressionmay play an important role in glioma progression.

### INPP4B overexpression restrains glioma cell growth, migration, and survival

**Figure 2:**
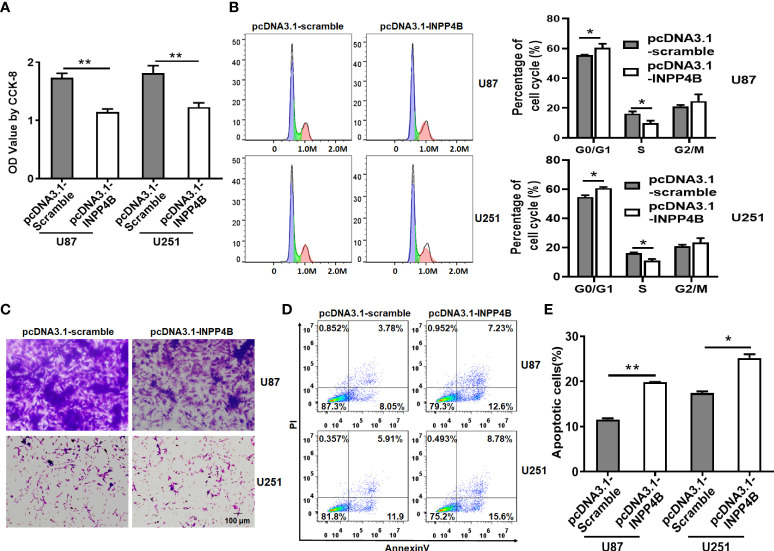
INPP4B overexpression inhibits the proliferation and migration of glioma cells, and increases their susceptibility to apoptosis. Human U87 and U251 glioma cells transfected with scramble (pCDNA3.1-Scramble) or INPP4B-overexpressing (pCDNA3.1-INPP4B) vector were assessed for **(A)** viability by CCK-8 assay; **(B)** cell growth by cell cycle assay; **(C)** migratory properties by transwell migration assay; and **(D, E)** survival to apoptosis, by Annexin V/PI staining and flow cytometry. Values represent the means ± SD (n = 3); *p < 0.05, **p < 0.01.

To test a potential link between INPP4B downregulation and glioma progression, we constructed an INPP4B-overexpressing vector (pcDNA3.1-INPP4B), using a scramble-overexpressing vector (pcDNA3.1-Scramble) as negative control. These vectors were transiently transfected into the human glioma cell line U87 and U251 (**Figure S1**). The effects of INPP4B on glioma cell growth and migration was tested *in vitro* by CCK-8, cell cycle and transwell assays. INPP4B overexpression obviously inhibited glioma cell proliferation and migration, while inducing G0/G1 cell cycle arrest (**Figures 2A–C**). Further, INPP4B overexpression resulted in a significant increase in the percentages of cells undergoing apoptosis (**Figures 2D, E**). Collectively, these findings demonstrated that *in vitro*, INPP4B suppresses glioma cell progression.

### INPP4B knockdown enhances glioma cell growth, migration, and survival

**Figure 3:**
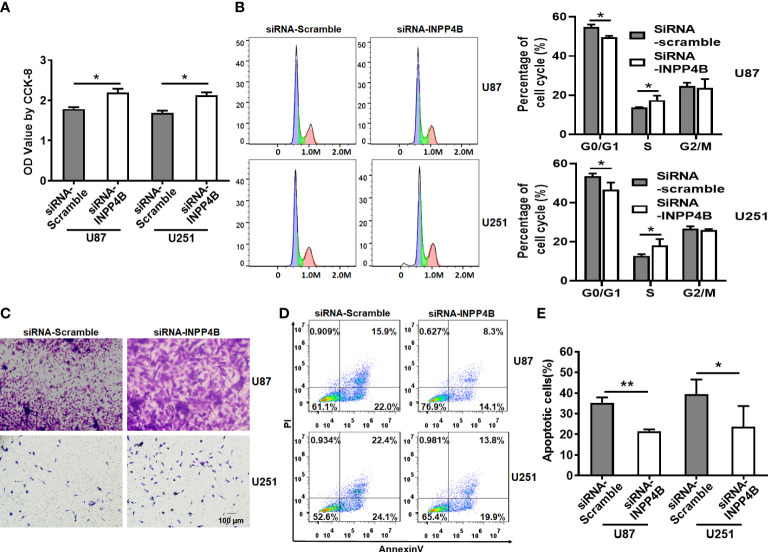
INPP4B knockdown promotes the proliferation and migration glioma cells and decreases their susceptibility to apoptosis. **(A)** CCK-8 assay on U87 and U251 cells transfected with siRNA-Scramble or siRNA-INPP4B. **(B)** Cell growth evaluated by cell cycle assay. **(C)** Migration of U87 and U251 cells after transfection with siRNA-Scramble or siRNA-INPP4B. **(D, E)** Effect of INPP4B knockdown on apoptosis in U87 and U251 cells, assessed by Annexin V/PI staining and flow cytometry. Values represent the means ± SD (n = 3). *p < 0.05, **p < 0.01.

To further demonstrate the beneficial role of INPP4B on restricting glioma growth, we sought to inhibit INPP4B expression in U87 and U251 cells. This was achieved by targeting INPP4B transcript with a specific siRNA (**Figure S2**). In contrast to INPP4B overexpression, INPP4B knockdown resulted in increased proliferation, migration of glioma cells, and reduced G0/G1 cell cycle arrest (**Figures 3A–C**). Additionally, INPP4B knockdown significantly reduced apoptosis in U87 and U251 cells, rendering these cells more resistant (**Figures 3D, E**). These results demonstrated that INPP4B downregulation may promote glioma progression *in vitro*.

### INPP4B negatively regulates PI3K/AKT signaling in glioma

**Figure 4:**
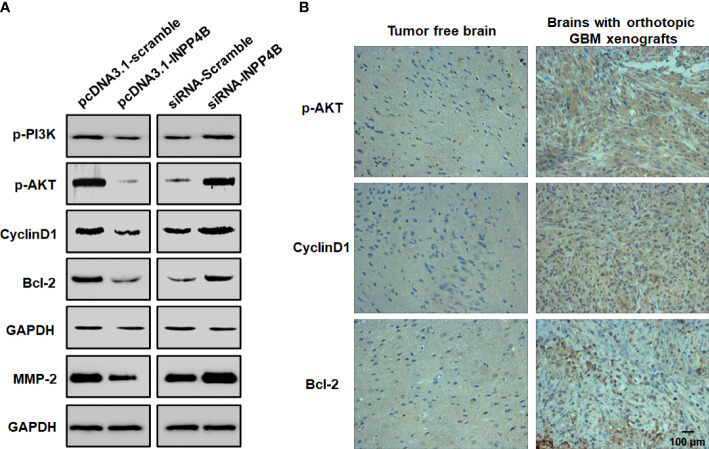
INPP4B negatively regulates PI3K/Akt signaling in glioma cells. **(A)** Quantification of p-PI3K, p-Akt, Cyclin D1, Bcl-2 and MMP2 proteins by western blot in glioma cells. **(B)** Representative IHC analysis of p-Akt, Cyclin D1 and Bcl-2 expression in mouse orthotopic glioma cells xenografts and in normal brain tissues. Values represent the means ± SD (n = 3). Scale bar, 100 μm.

INPP4B may regulate PI3K/AKT signaling in tumors (13). To further explore the potential mechanism whereby INPP4B inhibits glioma cell growth, we quantified phosphorylated PI3K and AKT, which represent two key factors in this signaling pathway. INPP4B overexpression in U87 cells markedly decreased the phosphorylation of PI3K and AKT. At the same time, proteins downstream of PI3K/AKT signaling, such as Cyclin D1, Bcl-2 and MMP2 were also decreased, whereas INPP4B knockdown had the opposite effects (**Figure 4A**). In the brain of orthotopic glioma mice, the expression of downstream targets of PI3K/AKT signaling that regulate GBC proliferation and apoptosis resistance, *e.g.*, Cyclin D1 and Bcl-2, was significantly increased compared with that in tumor-free brains (**Figure 4B**). Altogether, these observations suggest that INPP4B negatively regulates PI3K/AKT signaling in glioma.

### INPP4B overexpression reduces PD-L1 expression and T cell-suppression by glioma cells

**Figure 5:**
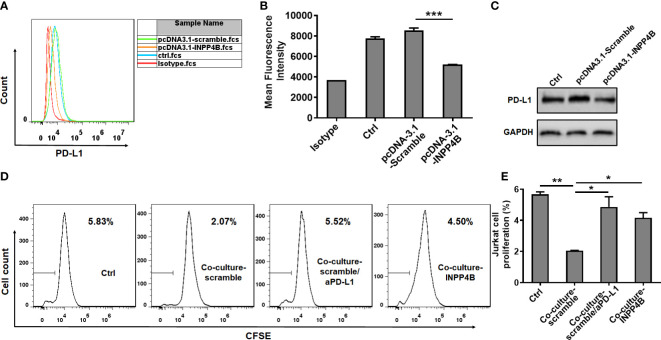
INPP4B overexpression reduces PD-L1 expression and T cell-suppression by glioma cells. U87 glioma cells transfected with scramble (pCDNA3.1-Scramble) or INPP4B-overexpressing (pCDNA3.1-INPP4B) vectors were assessed for PD-L1 expression by **(A, B)** flow cytometry or **(C)** western blot. **(D, E)** The proliferation of Jurkat cells was analyzed under four conditions (Jurkat cells alone, Jurkat cells co-cultured with U87 cells transfected with scramble [pCDNA3.1-Scramble], Jurkat cells co-cultured with U87 cells transfected with INPP4B-overexpressing [pCDNA3.1-INPP4B] vector, and Jurkat cells co-cultured with scramble-transfected U87 cells treated with anti-PD-L1 antibody [αPD-L1]). The percentage of cells that had undergone division (discrete peaks corresponding to CFSE dilution: gates on the histogram plots) was measured by flow cytometry. Values represent the means ± SD (n = 3); *p < 0.05, **p < 0.01, ***p < 0.0005.

To investigate the effect of INPP4B on PD-L1 expression, U87 glioma cells were transfected with pcDNA3.1-Scramble or pcDNA3.1-INPP4B for 48 h and PD-L1 expression was assessed by flow cytometry. PD-L1 expression appeared downregulated in the U87 cells transfected with pcDNA3.1-INPP4B (**Figures 5A, B**), which was confirmed by western blot analysis (**Figure 5C**). To further test the effect of INPP4B expression in glioma cells on their ability to inhibit T cell proliferation, after transfecting the U87 cells for 48 h with pcDNA3.1-Scramble or pcDNA3.1-INPP4B, CSFE-labeled Jurkat cells were added to the cultured and analysed for proliferation. The basal Jurkat cell proliferation without U87 cells was used as control. In co-culture with the glioma cells, Jurkat T cells proliferation was inhibited. However, when the U87 cells overexpressed INPP4B or when PD-L1 was blocked in the co-culture, Jurkat T cell proliferation was significantly higher, and almost restored to its basal level (**Figures 5D, E**). Altogether, these results showed that INPP4B overexpression restrains T cell-suppression by glioma cells.

### INPP4B overexpression restrains glioma cell proliferation, migration, and PD-L1 expression by down-regulating PI3K/AKT signaling

**Figure 6:**
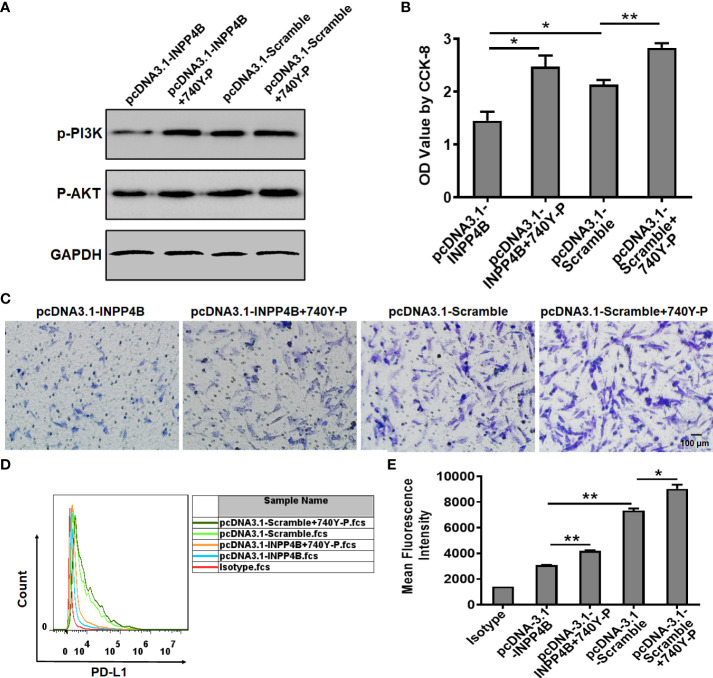
INPP4B overexpression restrains glioma cell proliferation, migration, and PD-L1 expression by down-regulating PI3K/AKT signaling. Effect of the PI3K/AKT activator 740Y-P on proliferation, migration, and PD-L1 expression in INPP4B-overexpressing U87 cells. **(A)** p-PI3K and p-AKT protein expression; **(B)** proliferation assessed by CCK-8 assay; **(C)** migratory properties assessed by transwell migration assay; and **(D, E)** PD-L1 expression assessed by flow cytometry. Values represent the means ± SD (n = 3); *p < 0.05, **p < 0.01.

In U87 cells, INPP4B overexpression reduced p-PI3K and p-AKT protein expression, while treatment with the PI3K/AKT activator 740 Y-P enhanced p-PI3K and p-AKT protein expression. These results suggested that the effect of 740 Y-P in U87 cells was specific and could be used for functional analysis (**Figure 6A**). INPP4B overexpression downregulated U87 cell proliferation, whereas 740 Y-P upregulated U87 cell proliferation at 48 h (**Figure 6B**). In addition, INPP4B overexpression downregulated U87 cell migration, whereas 740 Y-P had the opposite effect (**Figure 6C**). To investigate whether INPP4B restrained PD-L1 expression by glioma cells through interfering with PI3K/AKT signaling, U87 cells were transfected with pcDNA3.1-Scramble or pcDNA3.1-INPP4B for 48 h with or without 740 Y-P, and surface PD-L1 was analyzed by flow cytometry. While INPP4B overexpression downregulated PD-L1 expression, on the contrary, 740 Y-P up-regulated PD-L1 on U87 cells (**Figures 6D, E**). In short, INPP4B overexpression restrains glioma cell proliferation, migration, and PD-L1 expression by down-regulating PI3K/AKT signaling.

### INPP4B knockdown promotes glioma cell proliferation, migration, and PD-L1 expression by up-regulating PI3K/AKT signaling

**Figure 7:**
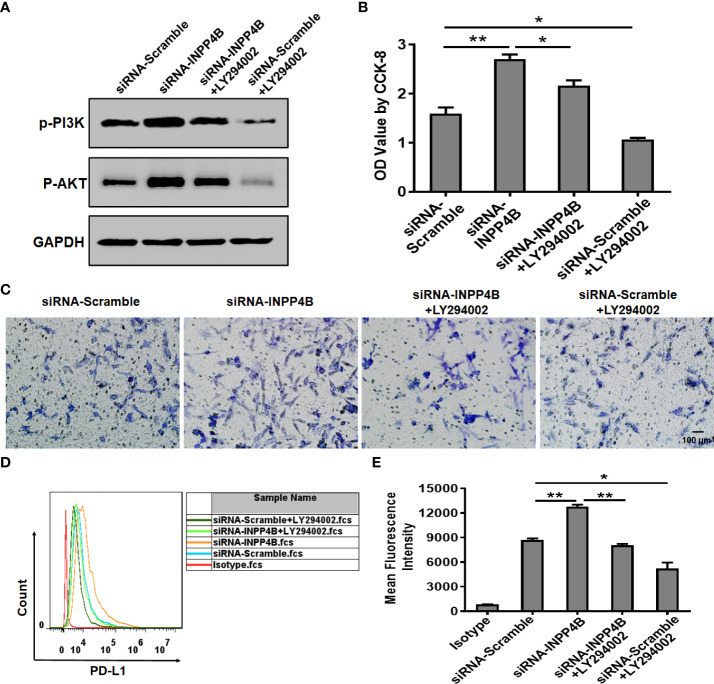
INPP4B knockdown promotes glioma cell proliferation, migration, and PD-L1 expression by up-regulating PI3K/AKT signaling. Effect of the PI3K/AKT inhibitor LY294002 on proliferation, migration, and PD-L1 expression in INPP4B-knockdown U87 cells. **(A)** p-PI3K and p-AKT protein expression; **(B)** proliferation assessed by CCK-8 assay; **(C)** migratory properties assessed by transwell migration assay; **(D, E)** PD-L1 expression assessed by flow cytometry. Values represent the means ± SD (n = 3); *p < 0.05, **p < 0.01.

In U87 cells, INPP4B knockdown promoted p-PI3K and p-AKT protein expression, while treatment with the PI3K/AKT inhibitor LY294002 reduced p-PI3K and p-AKT protein expression. These results suggested that the effect of LY294002 in U87 cells was specific and could be used for functional analysis (**Figure 7A**). INPP4B knockdown up-regulated U87 cell proliferation, whereas LY294002 downregulated U87 cell proliferation at 48 h (**Figure 7B**). In addition, INPP4B knockdown up-regulated U87 cell migration, whereas LY294002 had the opposite effect (**Figure 7C**). To investigate whether INPP4B restrained PD-L1 expression by glioma cells through interfering with PI3K/AKT signaling, U87 cells were transfected with siRNA-Scramble or siRNA-INPP4B for 48 h with or without LY294002, and surface PD-L1 was analyzed by flow cytometry. While INPP4B knockdown up-regulated PD-L1 expression, on the contrary, LY294002 down-regulated PD-L1 on U87 cells (**Figures 7D, E**). Thus, INPP4B knockdown promotes glioma cell proliferation, migration, and PD-L1 expression by up-regulating PI3K/AKT signaling.

## Discussion

The incidence of glioma is high, and surgery represents the first treatment choice. However, because of its high malignancy, fast growth rate, and especially high invasiveness, distinguishing tumor boundaries is highly challenging and sometimes impossible during most operations, even after radiotherapy and chemotherapy. Thus, glioma patients still face a very high recurrence rate. Since glioma is a serious threat to human health, exploring the mechanisms underlying its occurrence and development, as well as finding satisfactory therapies, are essential (2–4). Therefore, it is necessary to study glioma from the perspective of molecular biology.

INPP4B is a potential tumor suppressor gene; the main function of INPP4B is to inhibit the activation of the PI3K/AKT pathway, and thereby, the proliferation and survival of tumor cells. In some tumors, INPP4B acts as a tumor suppressor in similar ways as the protein PTEN. For example, in prostate cancer (28), the expression of INPP4B is regulated by androgens, which promote its expression in human prostate cells. In turn, INPP4B inhibits AKT phosphorylation, which inhibits malignant cell migration and invasiveness, and exerts a suppressive role on cancer progression. However, recent studies have contradicted this function and showed that INPP4B could promote tumor growth. That is, in colon cancer, INPP4B promotes cell proliferation through SGK3 phosphorylation of AKT (29). Since the function of INPP4B in glioma has not been reported, we sought to probe its suppressive or pro-tumoral effect on this malignancy.

Altered levels of INPP4B expression have been linked to cancer progression in various human tumor types (28–30). We provided evidence that INPP4B is expressed at low levels in glioma tissues and is negatively correlated with the pathological grade of glioma (**Figures 1A, B**, **Table S2**). These results suggest that INPP4B immunostaining may also be used as an immunomorphologic index to determine the pathological grade of glioma. The collection and analysis of clinical samples of patients’ tumors provide crucial information in cancer research (31). The abnormal expression from INPP4B we identified in clinical samples from glioma patients constitutes a valuable insight for future studies. Mouse orthotopic brain glioma models are important assets for the functional study of glioma (27). For this reason, we endeavored to establish a mouse orthotopic brain glioma model, which showed that INPP4B expression was lower in glioma tissues than in normal brain tissues (**Figure 1E**). Through the combined analysis of glioma patients’ samples, glioma cell lines and our mouse glioma model, we found that INPP4B is indeed under-expressed in gliomas, while it is highly expressed in normal astrocytes. It is well established that most gliomas originate from normal astrocytes (8, 9). Therefore, whether the abnormal expression of INPP4B contributes to the deterioration of astrocytes into glioma deserves further exploration. INPP4B suppresses cell invasiveness in prostate cancer (28) and hepatocellular carcinoma (30) but promotes colorectal cancer cell proliferation (29). Through overexpression and knockdown of INPP4B in U87 and U251 glioma cells we showed that INPP4B could inhibit the proliferation, migration, and apoptosis resistance of glioma cells (**Figures 2**, **3**). Thus, combined with previous findings in other tumors, our experiments demonstrate a tumor-specific effect of INPP4B, which may either inhibit or promote tumor progression through different pathways. As a major signal transduction pathway in tumor cells, the PI3K/AKT axis was shown to play an important role in the occurrence and development of glioma both *in vivo* and *in vitro*. With the technological developments of the molecular biology, this signaling pathway has become a new and increasingly important target for the treatment of glioma (14). In spite of a controversy regarding INPP4B and its function in tumorigenesis and metastasis, INPP4B plays a significant role in PI3K/Akt pathway-mediated tumorigenesis (30). Due to PtdIns(3,4)P2 and PIP3’s ability to recruit Akt to the plasma membrane, INPP4B is predicted to act as a tumor suppressor by inhibiting Akt recruitment, activation, and downstream PI3K signaling (32). We examined the role of PI3K/AKT and downstream regulatory proteins, *e.g.*, the proliferation-related protein cyclin D1 and the anti-apoptosis-related protein Bcl-2, by overexpressing or knocking down of INPP4B *in vitro* (**Figure 4A**). Moreover, we demonstrated for the first time that INPP4B negatively regulates PI3K/AKT signaling in orthotopic GBM xenotransplantation model *in vivo* (**Figure 4B**).

Immune escape in the context of malignant progression of glioma has become a hot topic of research for clinical tumor immunotherapy, in which the checkpoint pathway represented by the PD-L1/PD-1 axis is of great significance (31, 33, 34). Some studies have confirmed that different molecules belonging to this pathway are highly expressed in glioma but not in normal brain tissues and para-tumor tissues, which may be closely related to the clinicopathological features and prognosis of the patients. Previous studies have shown that loss of the tumor suppressor gene PTEN resulted in glioma’s immune resistance. In this process, the subsequent increase in PD-L1 expression played a key role (25). Meanwhile, our study found that INPP4B expression was negatively correlated with PD-L1 expression in glioma cells (**Figures 5A–C**). In addition, INPP4B overexpression in glioma cells could overcome the suppression they exerted on the proliferation of co-cultured T cells (**Figures 5D, E**). Thus, we presume that INPP4B loss results in glioma’s immune resistance, in which PD-L1 plays a key role, in a similar way as during PTEN loss. Although the mechanisms underlying immune checkpoint blockade are unclear, the PI3K/Akt pathway has been suggested to be closely related to the modulatory effects of the PD-1/PD-L1 pathway (35). Some oncogenic pathways contribute to PD-1/PD-L1 inhibitor-resistance. PTEN loss, which activates the PI3K/AKT pathway, induces PD-1 and PD-L1 inhibitor-resistance by promoting the release of anti-inflammatory cytokines that reduce infiltration and activation of CD8+ cytotoxic T cells in melanoma patients (36). In our experiment, we also found that INPP4B downregulated PD-L1 expression by inhibiting PI3K/AKT signaling (**Figures 6E, F**). We could infer that inhibiting the expression of INPP4B could activate the PI3K/AKT pathway, and thus upregulate PD-L1 expression, and ultimately, inhibit glioma’s immune escape.

In summary, here we present evidence that INPP4B expression is downregulated in glioma tissues and cells, and that INPP4B overexpression inhibits cell growth, migration, and apoptosis resistance of glioma cells. Further, INPP4B negatively regulates the expression of PD-L1, which likely compromises the ability of the glioma cells to escape immune surveillance and cytotoxicity. In addition, INPP4B may inhibit the proliferation, migration, apoptosis resistance and immune escape of glioma by negatively regulating PI3K/AKT signaling. Thus, INPP4B might be an attractive target for therapeutic intervention against glioma.

## Data availability statement

The original contributions presented in the study are included in the article/**Supplementary Material**. Further inquiries can be directed to the corresponding authors.

## Ethics statement

The use of human tissue was approved by the Ethics Committee of Hubei University of Medicine (2021-TH-001). The patients/participants provided their written informed consent to participate in this study. The animal study was reviewed and approved by the animal care committee of Hubei University of Medicine (2021-024).

## Author contributions

ZC and ZR designed the project. ZR collected cancer samples. XS wrote the first draft of the manuscript. XS and YC performed the animal and cellular experiments. XT and WZ performed the qPCRs, supervised the experiments, and participated in the revision of the manuscript. XYW and XHW performed statistical and data analyses, and participated in all the experiments. All authors contributed to the article and approved the submitted version.

## Funding

This work was supported by the National Natural Science Foundation of China (No. 82104205), the Natural Science Foundation of Hubei Province (No. 2021CFB143), and the Research Foundation for Talented Scholars of Hubei University of Medicine (No. 2019QDJZR06, 2020QDJZR001).

## Conflict of interest

The authors declare that the research was conducted in absence of commercial or financial relationships that could be construed as potential conflict of interest.

## Publisher’s note

All claims expressed in this article are solely those of the authors and do not necessarily represent those of their affiliated organizations, or those of the publisher, the editors and the reviewers. Any product that may be evaluated in this article, or claim that may be made by its manufacturer, is not guaranteed or endorsed by the publisher.
