# Supplementary material for: INPP4B inhibits glioma cell proliferation and immune escape via inhibition of the PI3K/AKT signaling pathway
Source: Front Oncol. 2022 Sep 6;12:983537. doi: 10.3389/fonc.2022.983537 (PMC9487419; doi:10.3389/fonc.2022.983537)
Supplement: Supplementary file 1 [file DataSheet_1.pdf]

Supplementary Material

Contents

Table S1: INPP4B expression is significantly diminished in glioma tissues.

Table S2: Correlations between INPP4B expression and different clinicopathological factors in 25 glioma patients.

Figure S1: pcDNA3.1-INPP4B, pcDNA3.1-Scramble, pcDNA3.1 were transiently transfected into the human glioma cell line U87.

Figure S2: Figure S3. siRNA-Scramble or siRNA-INPP4B were transiently transfected into the human glioma cell line U87.

Table S1. INPP4B expression is significantly diminished in glioma tissues.

**Grade**	**Cases**	**INPP4B expression**			***P*-Value**
		**Negative**	**Positive**	**Positive rate**	
Normal	20	6	14	70%	0.0234
Glioma	25	16	9	36%	

Note: * indicates $P < 0.05$, as assessed by Pearson chi-square test.

Table S2. Correlations between INPP4B expression and different clinicopathological factors in 25 glioma patients.

Characteristics	Total (N)	INPP4B expression		*P*-Value
		Negative	Positive	
**Gender**				
Male	10	6	4	>0.99
Female	15	10	5	
**Age**				
≤ 59	4	3	1	>0.99
> 59	21	13	8	
**Grade**				
Grade I–II	12	5	7	0.0414 *
Grade III–IV	13	11	2	

Note: * indicates $P < 0.05$, as assessed by Fischer's test.

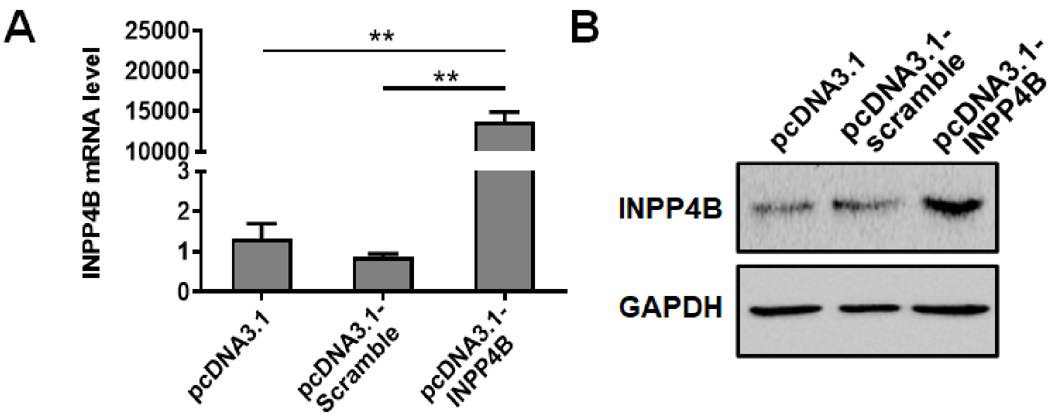

Figure S1. pcDNA3.1-INPP4B, pcDNA3.1-Scramble, pcDNA3.1 were transiently transfected into the human glioma cell line U87. Expression of INPP4B in U87 cell assessed by qRT-PCR **(A)** and western blot **(B)** after transfection with empty vector (pCDNA3.1), pCDNA3.1-INPP4B or pCDNA3.1-Scramble. Values represent the means ± SD (n = 3). $^{**}p < 0.01$.

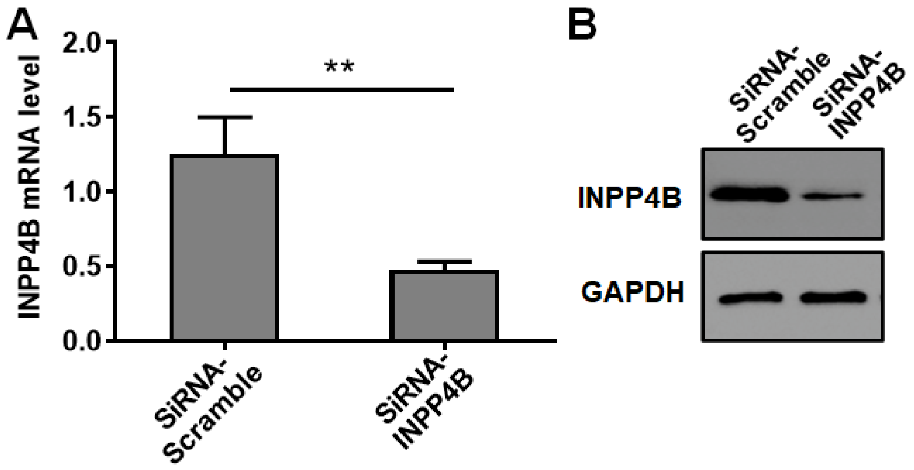

Figure S2. siRNA-Scramble or siRNA-INPP4B were transiently transfected into the human glioma cell line U87. The expression of INPP4B in U87 cells was assessed by qRT-PCR **(A)** and western blot **(B)** after transfection with siRNA-Scramble or siRNA-INPP4B. Values represent the means ± SD ($n = 3$, ***p* < 0.01).
